# B-Line Detection and Localization in Lung Ultrasound Videos Using Spatiotemporal Attention

**DOI:** 10.3390/app112411697

**Published:** 2021-12-09

**Authors:** Hamideh Kerdegari, Nhat Tran Huy Phung, Angela McBride, Luigi Pisani, Hao Van Nguyen, Thuy Bich Duong, Reza Razavi, Louise Thwaites, Sophie Yacoub, Alberto Gomez, VITAL Consortium

**Affiliations:** 1 School of Biomedical Engineering & Imaging Sciences, King’s College London, London SE1 7EU, UK; 2 Oxford University Clinical Research Unit (OUCRU), Ho Chi Minh City 700000, Vietnam; 3 Mahidol Oxford Tropical Medicine Research Unit (MORU), Bangkok 10400, Thailand; 4 Hospital of Tropical Diseases, Ho Chi Minh City 700000, Vietnam

**Keywords:** lung ultrasound (LUS) imaging, b-lines, spatiotemporal attention, classification, video analysis

## Abstract

The presence of B-line artefacts, the main artefact reflecting lung abnormalities in dengue patients, is often assessed using lung ultrasound (LUS) imaging. Inspired by human visual attention that enables us to process videos efficiently by paying attention to where and when it is required, we propose a spatiotemporal attention mechanism for B-line detection in LUS videos. The spatial attention allows the model to focus on the most task relevant parts of the image by learning a saliency map. The temporal attention generates an attention score for each attended frame to identify the most relevant frames from an input video. Our model not only identifies videos where B-lines show, but also localizes, within those videos, B-line related features both spatially and temporally, despite being trained in a weakly-supervised manner. We evaluate our approach on a LUS video dataset collected from severe dengue patients in a resource-limited hospital, assessing the B-line detection rate and the model’s ability to localize discriminative B-line regions spatially and B-line frames temporally. Experimental results demonstrate the efficacy of our approach for classifying B-line videos with an *F*1 score of up to 83.2% and localizing the most salient B-line regions both spatially and temporally with a correlation coefficient of 0.67 and an IoU of 69.7%, respectively.

## 1 Introduction

Dengue is a viral disease that can transmit to humans through the bites of an infected female Aedes genus mosquito [1]. Dengue can cause a wide range of symptoms from a mild febrile illness through to severe and life threatening manifestations such as shock, bleeding and organ dysfunction [2]. A hallmark feature of severe dengue is increased capillary permeability causing plasma leakage, which manifests as intravascular volume depletion and fluid accumulation such as pleural effusions, pulmonary edema and ascites [3]. Therefore, accurate and dynamic monitoring of fluid leak into the lungs of patients with severe dengue admitted to ICU is critical for optimal care.

**Figure 1:**
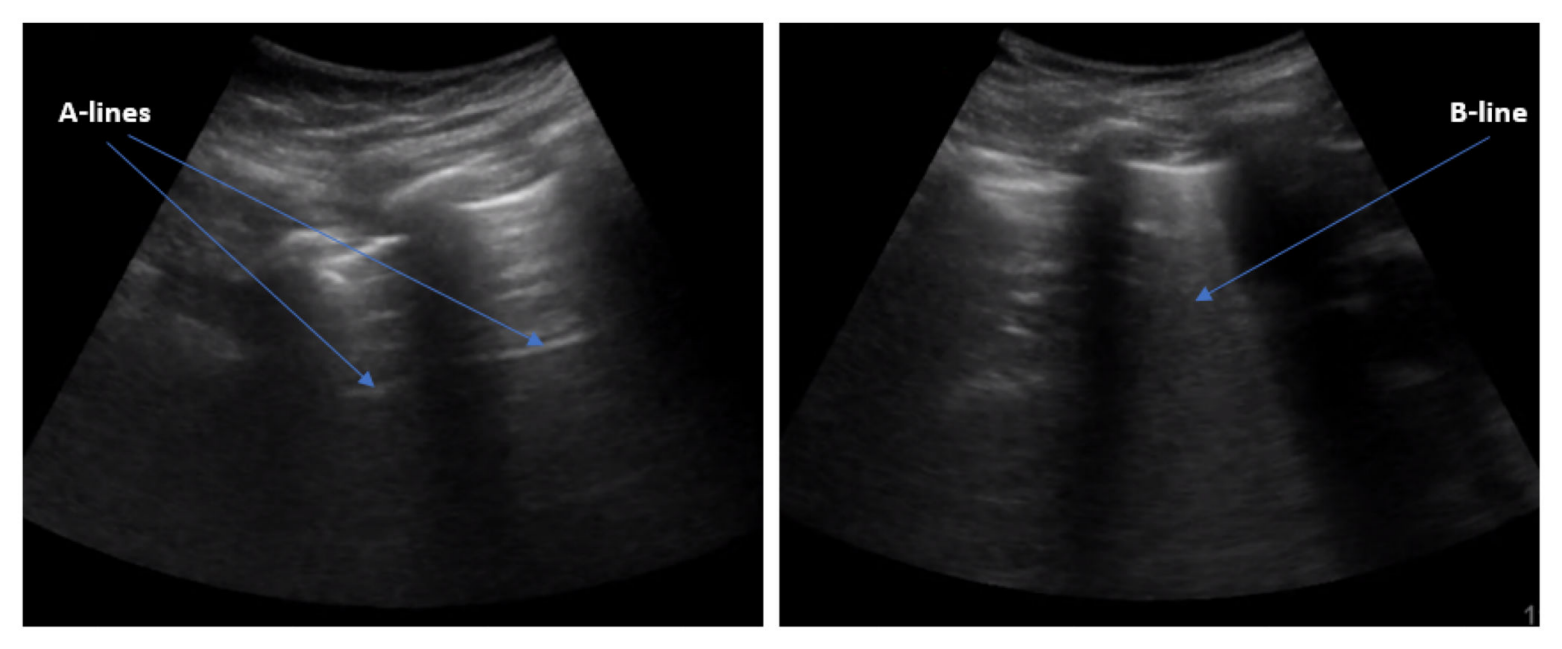
Sample LUS images. (**Left**): A healthy lung containing several A-line artefacts and, (**Right**): A dengue patient’s lung showing a B-line artefact as a result of fluid leakage into the lung.

Lung ultrasound (LUS) imaging is a fast, portable and safe imaging technique, employed as a reference modality in intensive care units (ICU) for rapid real-time lung assessment. Specifically, LUS imaging captures image artefacts such as B-lines, which are caused by fluid, that indicate a pulmonary abnormality such as edema and effusions [4]. To this end, LUS imaging can be used to assess fluid leakage into the lung through B-lines. As shown in Figure 1, right, B-lines are bright lines expanding from the lung surface distally following the direction of sound waves’ propagation. These lines appear and disappear from the ultrasound images during the respiratory cycle, and are only found in some regions of the affected lung [5]. Consequently, identification of these lines by visual inspection is a challenging task. Even more so for less experienced sonographers, this involves first identifying the frame where artefacts are visible, and second, determining whether such artefacts are B-lines or not.

Recently, automatic image analysis using deep learning (DL) methods have shown promise for various tasks such as the classification, reconstruction, and segmentation of tissues in ultrasound images [6,7]. Applied to LUS, classification models could assist automate problems such as B-line detection. Over the last few years, attention models have been used for tasks where interpretation or explanation requires only a small portion of the image [8], video [9] or sentence [10]. These models have also contributed to the model’s interpretability, by visualizing regions that the model attends to solve a specific task. In particular, in B-line detection from LUS videos, an attention mechanism can help localize where and when in the video the B-line features are.

### Contributions

**Figure 2:**
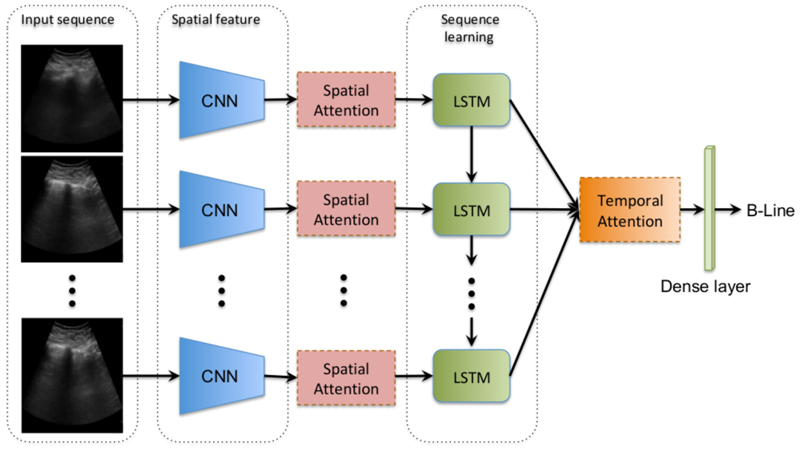
The proposed architecture for LUS B-line detection and spatiotemporal localization. This model consists of a spatial feature extraction module (CNN layers), followed by a spatial attention network, then a bidirectional LSTM, and a temporal attention module. The parameters of each layer and module are detailed in the text.

In this paper, we propose a novel B-line classification and localization method for a lung ultrasound (LUS) video B-line recognition to address the aforementioned challenges. Our method uses a spatiotemporal attention mechanism that consists of a spatial component and a temporal component as shown in Figure 2. This work extends our previous work [11,12] on the temporal localization of B-lines. The novel contributions of this work are four-fold: (1) A spatial attention mechanism for B-line region detection; (2) A novel evaluation metric for B-line spatial localization; and (3) An investigation of the effect of different LUS video lengths on model performance during the training and testing phases; and finally, (4) we qualitatively and quantitatively show that our spatiotemporal attention is able to localize B-line regions and frames, despite being trained in a weakly-supervised manner.

## 2 Related Work

Being an artefact-focused application, LUS has historically received far less attention than other ultrasound applications that focus on anatomical imaging. However, precisely because human interpretation is difficult, and because LUS relies in the analysis of image patterns, advances in deep learning which have achieved state-of-the-art performance in many image detection and classification tasks [13] can be very useful for LUS. The recent COVID-19 pandemic has motivated a significant increase in lung imaging research, and most existing work in automated LUS analysis is related to COVID-19. Related work can be broadly categorized in either detection of artefacts, such as B-lines or the localisation and segmentation of lung lesions.

The first category involves techniques that address the classification of LUS frames into B-line frames or not. Although most lung imaging studies use CT [14] and X-ray [15] images, recently some studies applied ultrasound imaging to assist the automatic detection of lung artefacts. For example, [16] employed a convolutional neural network (CNN) classifier on LUS images with B-lines of different etiologies to accurately distinguish COVID-19 from similar pathology, reporting an *F*1 score of 0.8, 0.75 and 0.81 for COVID, no COVID and HPE, respectively. Another study [6] proposed a method for the classification and weakly-supervised localization of LUS B-lines in COVID-19 patients, to an accuracy in-vivo of 0.83 to 0.89. Abnormalities in LUS images were recognised using a fully convolutional network followed by class activation maps (CAMs) [17] to generate a weakly-supervised segmentation of the images. Differently, a spatial transformer network was used by [18] for weakly-supervised localization of COVID-19 related artefacts, reporting a maximum *F*1 score of 0.71. Further, they predicted the presence of COVID-19 related artefacts and disease severity using an ordinal regression. Kulhare et al. [19] employed a single-shot CNN to predict bounding boxes for B-line artefacts, reporting a sensitivity/specificity of 0.88/0.93. Because all the above techniques apply the processing task in individual frames, they require prior retrieval of the relevant frame to be processed, either manually or using a method to extract candidate frames. Applying the processing task to every single frame to process an entire clip can be very sensitive to false positives. In our previous work [11], of which this paper is an extension, we proposed a temporal attention-based convolutional+LSTM model capable of detecting the B-line artefacts and localizing them within LUS videos, allowing us to exploit spatiotemporal features to classify the video and leveraging the temporal attention mechanism to identify the frame where the most salient features are.

The second category focuses on localising the image lesion, by using attention mechanisms and segmentation methods. Most of this work was carried out on lung images acquired from other modalities such as CT and X-ray. Chen et al. [14] proposed a residual attention U-Net for the multi-class segmentation of chest CT images from COVID-19 patients. A similar approach was employed for X-ray lung segmentation of pneumonia [15]. A 3D CNN network with online attention refinement and a dual-sampling strategy was developed by [20] to differentiate COVID-19 artefacts from pneumonia in chest CT images. Liu et al. [21] proposed a lesion-attention deep neural network (LA-DNN) that performs binary classification of the presence of COVID-19 and, in addition, five lesion classification with multi-label attention. Zhao et al. [22] proposed a dilated dual attention U-Net (D2A U-Net) for COVID-19 lesion segmentation in CT images. The dilated convolution module in a model decoder refines the decoding process and improves segmentation accuracy. Further, a dual attention mechanism, which is inserted to the skip connection and model decoder is utilized to refine feature maps and therefore reduce the semantic gap between different levels of the model. A U-Net based COVID-19 CT images segmentation network using an attention mechanism was proposed by [23]. They incorporated a spatial and a channel attention mechanism to a U-Net architecture to capture rich contextual relationships for better feature representation and, consequently, to enhance the network performance.

Previous work has used attention and CAMs to find the region in the image where the lesion was. Our proposed method can localize B-line regions in LUS videos both spatially and temporally despite being trained in a weakly-supervised fashion. We leverage temporal analysis networks, and use spatiotemporal attention to find the most important frames (i.e., B-line frames) and localize B-line regions within a video. Indeed, the ability to detect B-line frames in the LUS video is necessary for clinical application as B-line artefacts appear at arbitrary frames within an LUS video. Furthermore, detecting B-line regions in the LUS video along with the B-line frames would be the first step towards the quantification of the severity of the disease.

## 3 Methods

Our model is a recurrent neural network (RNN) that aggregates a frame-based convolutional feature extractor across the video to detect LUS B-line artefacts. As shown in Figure 2, the model architecture has four components: (1) convolutional frame feature extraction network (CNN); (2) spatial attention mechanism; (3) bidirectional long short-term memory (LSTM) network; and (4) temporal attention mechanism. The convolutional features are attended over both spatially, in each frame, and subsequently temporally for the entire video sequence. Both employed attentions are soft attentions which means the effective final representation at time *t* is a spatiotemporally weighted aggregation of convolutional features across the video.

### 3.1 Convolutional Frame Feature Extraction Network

**Figure 3:**
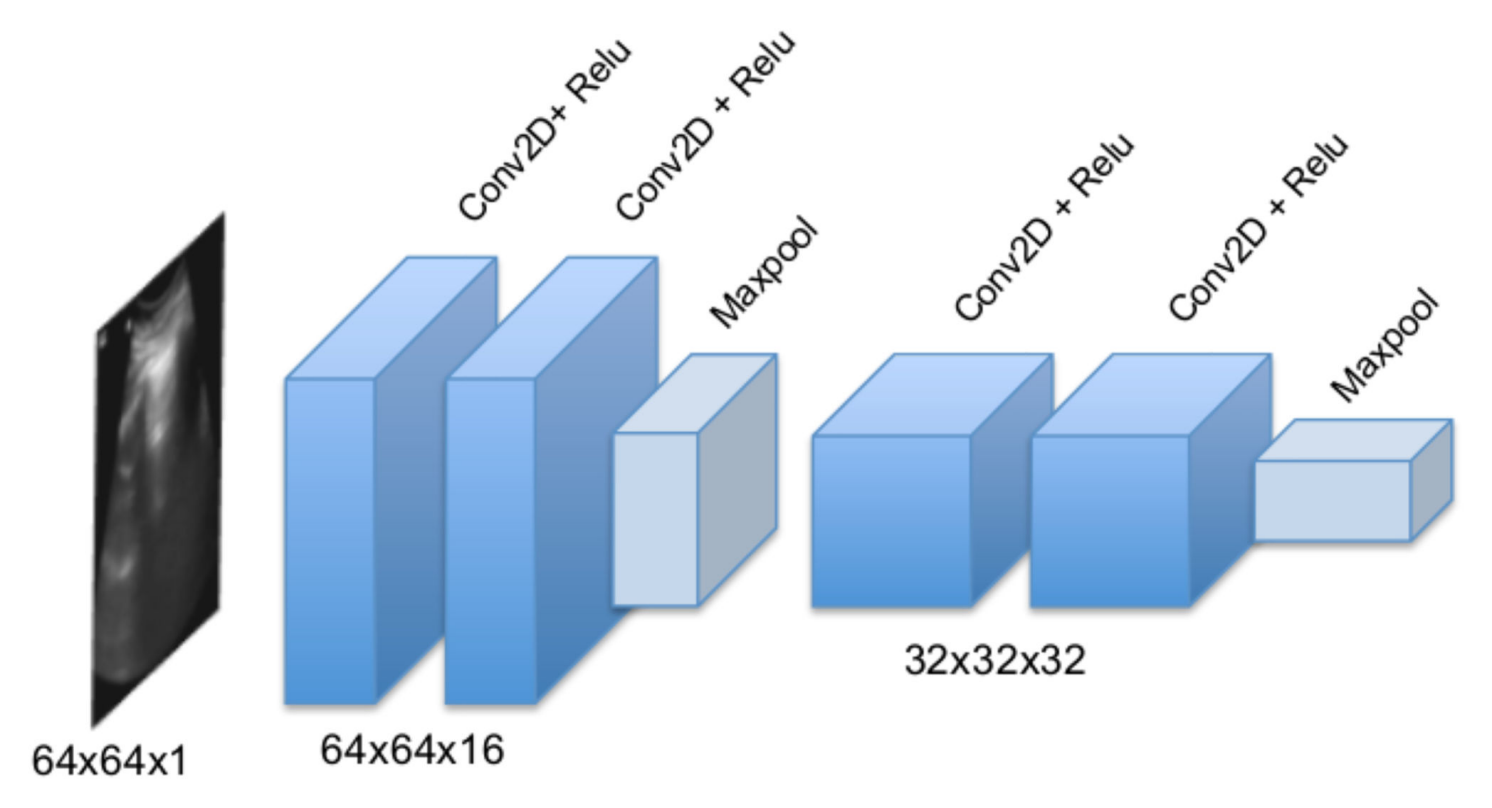
The CNN architecture of the proposed model. It consists of four convolution layers with Relu activation function and; Maxpooling followed by the second and forth convolution layer.

The input *X* to the model is a sequence of *n* frames, resized to 64 × 64 pixels, *X* = (*x*_0_, …, *x*_*n*_), *X* ∈ ℝ^*D*^. This input is fed to the CNN part. The CNN architecture is shown in Figure 3. This part consists of four layers of convolution with ReLU activation, and two max-poolings. Each convolution filter uses 3 × 3 kernels with unit stride, and 16 and 32 output channels, respectively. More accurate spatial feature extractors, for instance, networks with more parameters such as ResNet-152 [24] and DenseNet [25], will likely lead to a better overall performance. However, we keep the features relatively simple because the primary purpose in this paper is to prove the efficacy of our spatiotemporal attention mechanism.

### 3.2 Spatial Attention Mechanism

**Figure 4:**
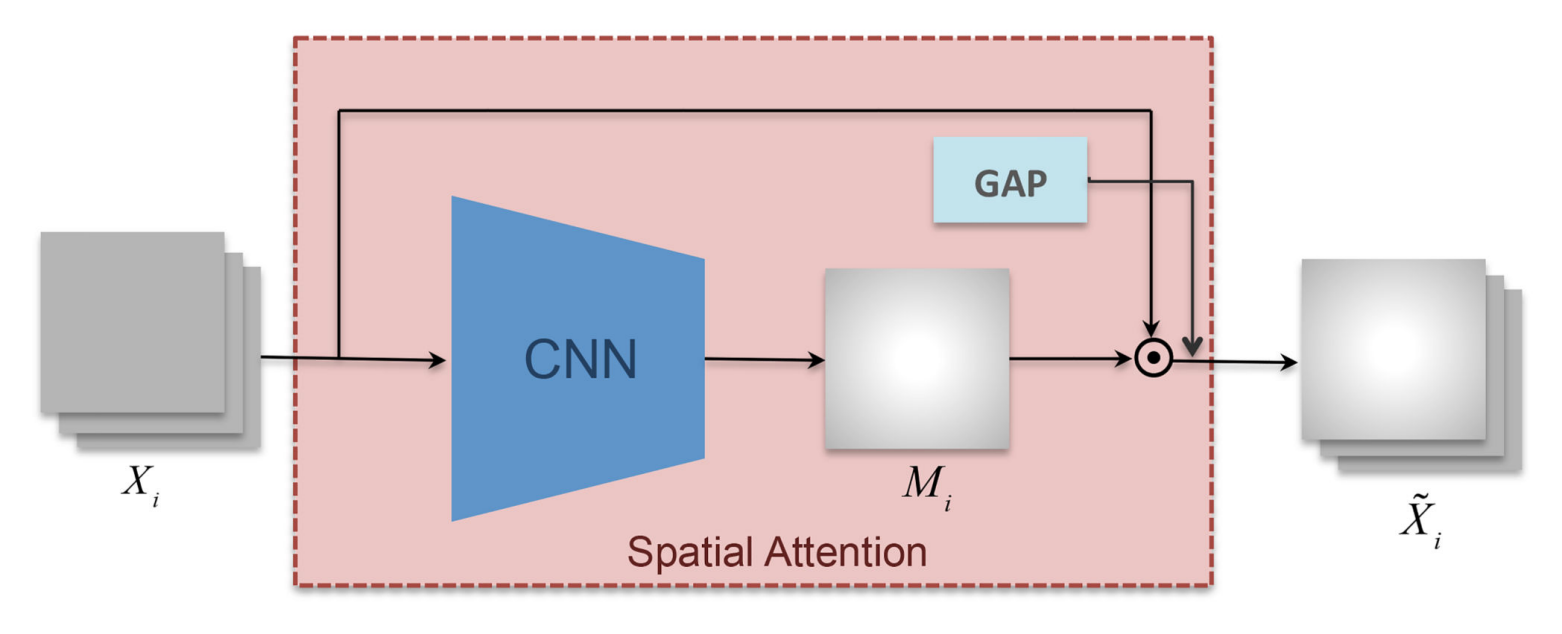
Spatial attention module. Several layers of convolutional networks (for details see Table 1) are used to learn the importance mask *M_i_* for the input image feature *X_i_*, the output is the element-wise multiplication ${\tilde{X}}_{i}=X_{i}⊙M_{i},$. GAP: Global Average Pooling.

Spatial attention allows us to focus on regions of the frames that contribute to a higher classification accuracy; in this case, to B-line classification, hence it should be able to focus on B-line artefacts. The feature map output by the CNN network (explained in the previous section) is passed as an input to our spatial attention module. Attention is achieved by learning a map *M*_*i*_ that masks the image feature *X*_*i*_ of the *i*th frame to obtain attended image features, as in [26]:

(1) $${\tilde{X}}_{i}=X_{i}⊙M_{i},$$

where ⊙ denotes element-wise multiplication; for 1 ≤ *i* ≤ *n, n* is the number of frames, and each entry of *M* lies in [0, 1]. This operation attenuates certain regions of the feature map based on their estimated importance. Figure 4 illustrates the spatial attention mechanism.

In order to learn the importance mask *M*_*i*_, we apply three convolutional layers (architecture details are in Table 1) on the image feature *X*_*i*_. Finally, the attended image feature ${\tilde{X}}_{i}$ represents a region highlighted (or suppressed) version of *X*_*i*_ that is globally pooled, hence delegating any ability to resolve features spatially to the attention mask.

### 3.3 Bidirectional LSTM and Temporal Attention

The LSTM and temporal attention parts are taken from our previous work in [11]. For completeness, a summary is presented here but the interested reader can refer to the original paper for further details.

The spatial attention output, a 1 × 1 feature map with 32 channels, is passed as an input to the LSTM to extract temporal information. The generated LSTM outputs are then sent over to the temporal attention network to produce the attention score for each attended frame in the LUS video.

The temporal attention mechanism proposed for neural machine translation [27] was adapted to our application as described in [11]. Essentially, frame-wise importance attention weights are used to multiply the LSTM feature vector output; therefore, effectively learning which frame of the LUS video to pay attention to. The temporal feature vector, which is attention-weighted, is now averaged over time and used by a fully connected layer for LUS video classification. The code we used to train and evaluate our models is available at https://github.com/hamidehkerdegari/Spatiotemporal-Attention-for-B-line-Detection (accessed on 1 December 2021).

## 4 Materials and Experimental Setup

### 4.1 Data Collection and Annotation

The LUS scans were performed using a Sonosite M-Turbo machine (Fujifilm Sonosite, Inc., Bothell, WA, USA) with a low–medium frequency (3.5–5 MHz) convex probe by qualified sonographers. A standardised operating procedure based on the Kigali ARDS protocol [28] (assessment for B-lines [29,30], consolidation and pleural effusion) was performed at six points on each side of the chest (two anterior, two lateral and two posterolateral).

**Figure 5:**
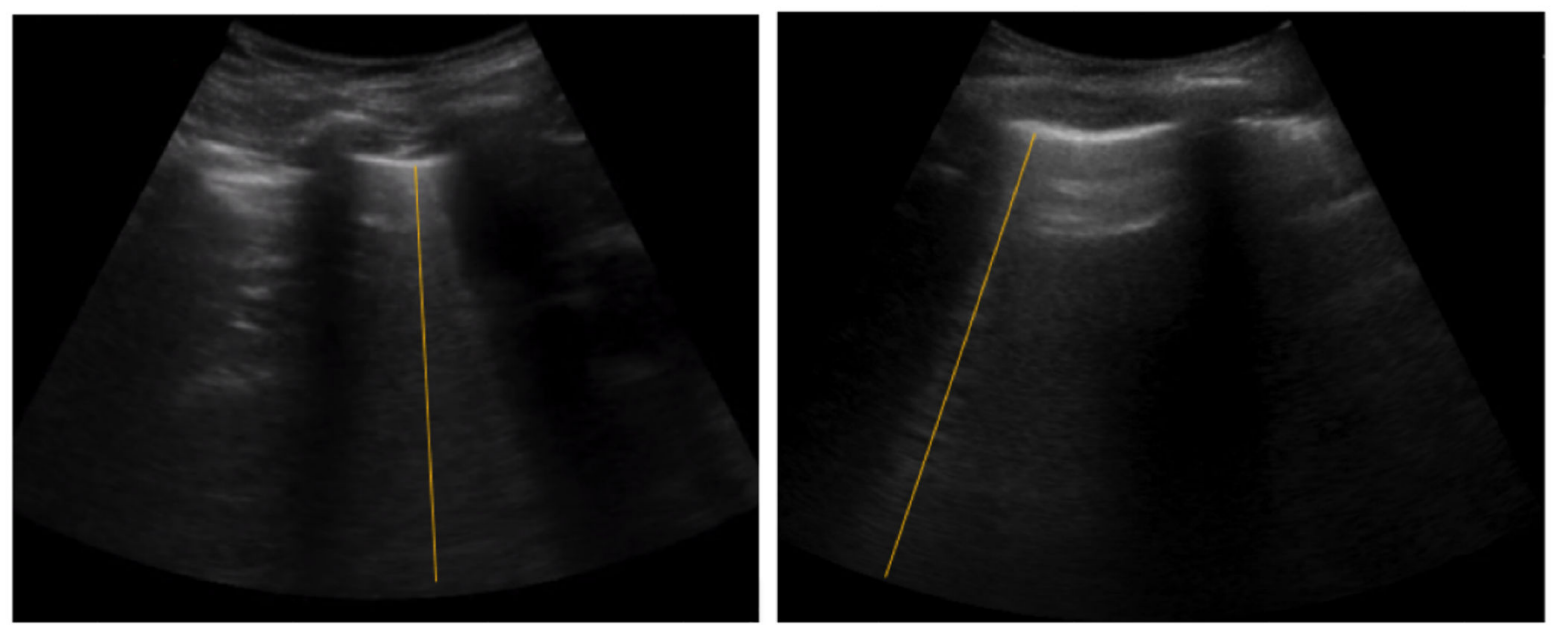
Examples of B-line regions annotation used for spatial attention task. A straight yellow line was drawn on the B-line region extending from the surface of the lung distally following the direction of propagation of the sound waves.

Data were collected from 60 Dengue patients at the Hospital of Tropical Diseases (HTD) in Ho Chi Minh City, Vietnam. All patients were recruited following approved institutional ethics and after informed consent, and in agreement with the Declaration of Helsinki. This LUS video dataset contains approximately five hours B-line and non-B-line video data with video resolution of 640 × 480 pixels and a frame rate of 30 fps. Then, each four-second clip was saved in Audio Video Interleave (AVI) format, fully anonymised through masking, and cropped to a square-shaped region around the ultrasound frustum, as shown in Figure 5.

These anonymised LUS video clips were annotated by a qualified clinician using the VGG annotation tool [31]. During the annotation procedure, a label that is either B-line or non-B-line was assigned to each video clip. Then, the B-line frames in the B-line videos were annotated for the temporal localization task using the proposed temporal attention mechanism. Furthermore, the B-line regions were annotated using a straight line as shown in Figure 5 for the spatial localization task with our spatial attention mechanism. The annotation outputs were saved in Java Script Object Notation (JSON): https://www.json.org/ (accessed on 1 December 2021) format to be used as ground truth.

For training the model, we converted each four-second video clip into shorter clips of one second with 20% overlap between consecutive frames in the video. The overview of our LUS video dataset is presented in Table 2.

### 4.2 Implementation Details

Our model was implemented in Python, using Keras with Tensorflow. The model was trained using an Adam optimizer, with a learning rate set to 10^−6^ and a batch size of 25. Dropout layers with a probability of 0.2 were also used to help regularize the training process. Input videos were resampled to 64 × 64 pixels during the training stage. We augmented our dataset by adding horizontally-flipped frames to the training data. We carried out 5-fold cross validation and trained the network for 150 epochs, after all models had converged.

### 4.3 Evaluation Metrics

As an evaluation metric for the B-line classification task, we used the harmonic mean of precision and recall expressed as the *F*1 score in percentages.

(2) $$F1=2\times \frac{Precision\times Recall}{Precision+Recall}\times 100.$$

Intersection Over Union (IoU) of the predicted and ground truth temporal labels was used as the temporal attention mechanism error metric.

**Figure 6:**
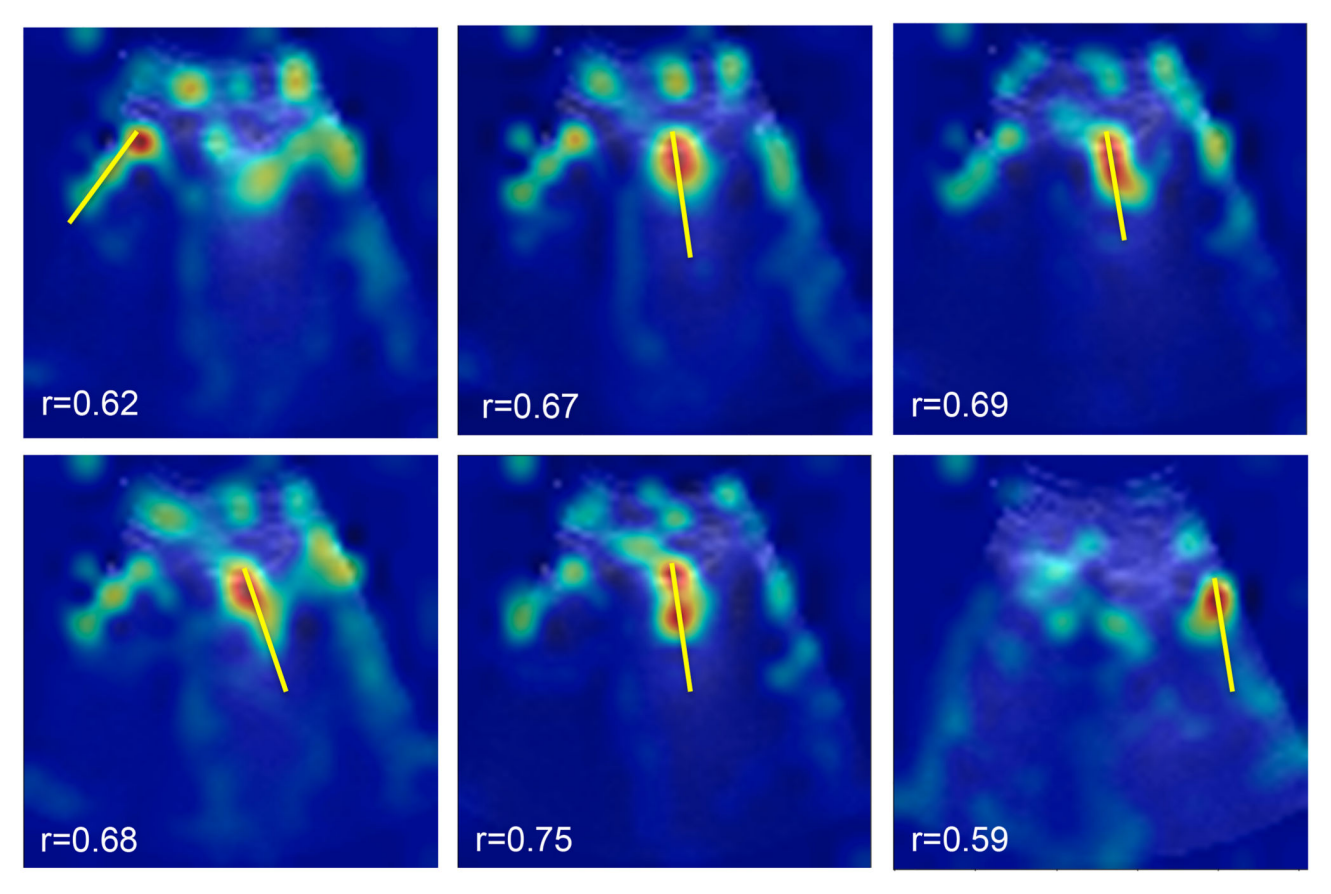
Examples of spatial attention map for B-line localization task. Our spatial attention module can automatically highlight B-line regions (red areas) and avoid irrelevant regions corresponding to no-B-line regions or background. Yellow straight lines represent ground truth. Correlation coefficient values (*r*) are presented at the bottom of each attention map.

The Pearson correlation coefficient [32] was applied as a metric for the B-line spatial localization task. The Pearson correlation coefficient, *r*, is a measure of the linear correlation between two datasets which are B-line region annotation (shown in Figure 5) and generated attention map (shown in Figure 6). This measure was calculated as follows:

(3) $$r=\frac{\sum_{i=1}^{n}\left(x_{i}-\bar{x}\right)\left(y_{i}-\bar{y}\right)}{\sqrt{\sum_{i=1}^{n}{\left(x_{i}-\bar{x}\right)}^{2}}\sqrt{\sum_{i=1}^{n}{\left(y_{i}-\bar{y}\right)}^{2}}}.$$

This equation shows how much the two datasets, *x* = [*x*_1_*x*_2_…*x*_*n*_] and *y* = [*y*_1_*y*_2_…*y*_*n*_], vary together compared to how much they vary separately. The magnitude of the correlation coefficient indicates the strength of the correlation and the sign indicates whether the correlation is positive or negative.

## 5 Experiment Results

We carried out five types of experiments: first, experiments to evaluate our proposed method on the B-line classification task using the LUS video dataset described earlier; second, experiments to evaluate the spatial attention mechanism on the B-line spatial localization task; third, experiments to evaluate the temporal attention mechanism on the B-line temporal localization task; fourth, experiments to investigate the effect of different video lengths on model performance during the training and testing phase; and fifth, several ablation experiments were conducted to evaluate the performance of components presented in our model. Each of these experiments is described in turn.

### 5.1 B-Line Classification

We compared three model architectures to investigate the potential benefit of using temporal information in the B-line detection task. Our baseline model (C2D+S+T) has 2D convolutions in the initial CNN subnet followed by spatial and temporal attention modules without LSTM. The second model (C3D+S+T) is a model with 3D convolutions in the CNN subnet followed by spatial and temporal attention and no LSTM. The last model (C2D+S+LSTM+T) has 2D CNN followed by spatial attention, LSTM and temporal attention.

Table 3 shows the classification results on our LUS video dataset. The performance of the C2D+S+T model is least (59.9%), because without the LSTM model it cannot model the temporal part of the video. With the C3D+S+T model, the performance improves (78.3%), which indicates the ability of the C3D network for modelling the data temporal aspect but with a shorter context span over time compared to LSTM. However, when LSTM is added to the C2D model in C2D+S+LSTM+T, the *F*1 score increases (83.2%), which indicates the importance of the LSTM model for the B-line classification as it considers long temporal progression of the LUS video data.

### 5.2 Spatial B-Line Localization

The spatial localization mechanism highlights regions in LUS frames, which contribute most to the detection task. Here, we verify the hypothesis that such regions are indeed the location of B-lines.

Evaluating spatial localization is a challenging task that heavily depends on the available annotations. For instance, Meng et al. [33] applied thresholding on the saliency mask for activity recognition in videos with bounding box annotations. The tightest bounding box that contains the thresholded saliency map is considered the predicted localization bounding box for each frame. Then, the predicted localization boxes were compared with the ground truth bounding boxes at different intersection over union (IoU) levels. Differently, in our study, B-line regions were annotated with a straight line as shown in Figure 5. Further, we noticed that our attention maps tend to highlight the beginning of the B-line as shown in Figure 6, which may correlate with the lung structure generating the B-line, yielding a correct localization even if the attention does not span over the entire length of the artefact. As a result, thresholding and IoU were not suitable for our evaluation.

**Figure 7:**
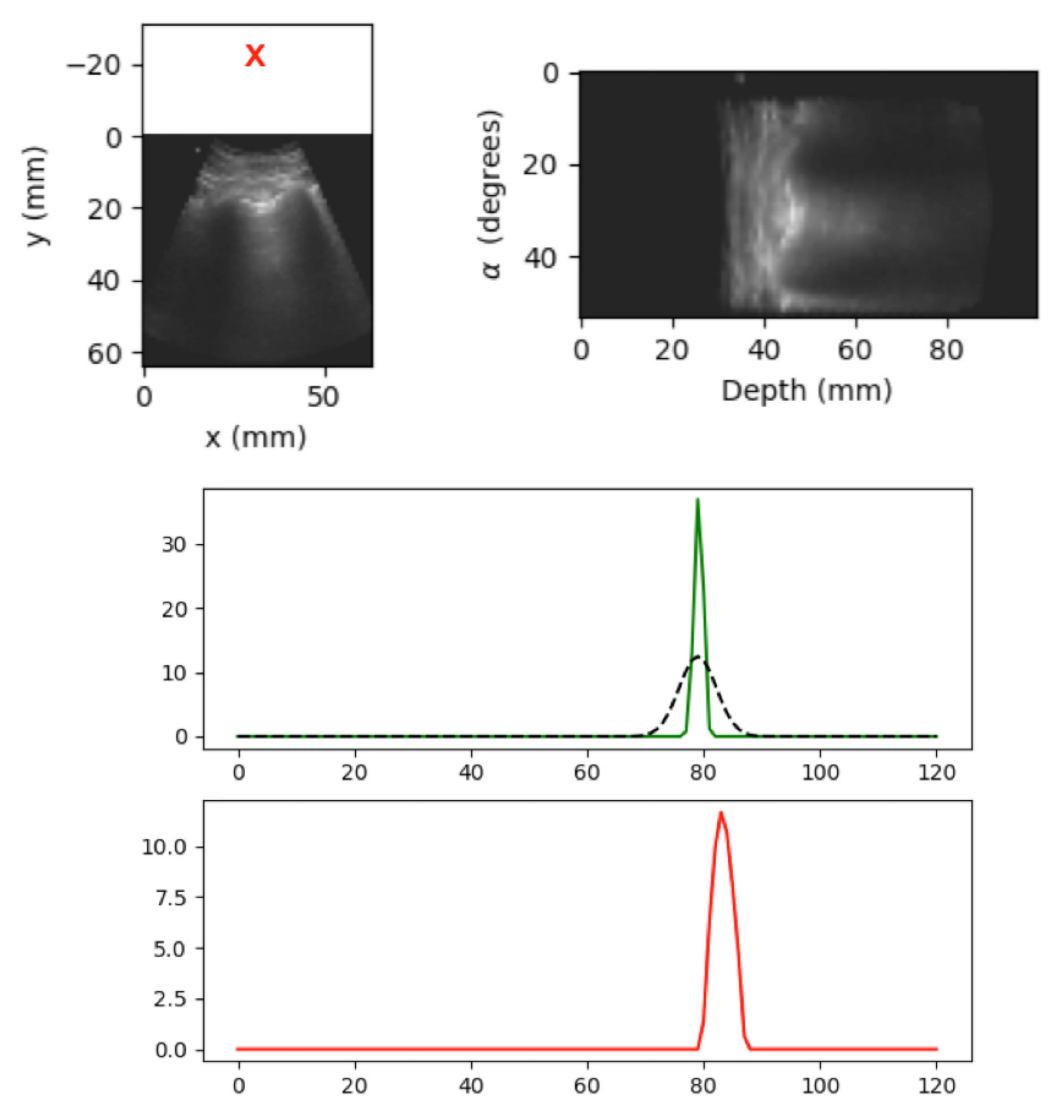
**Top**: An example of polar coordinates applied to a sample B-line frame; the red cross shows the beam source. **Center**: The generated 1-dimensional diagram showing it’s related ground truth (green line, the black line is normal distribution), and **Bottom**: attention map values (red line) across the coordinates. In this example, the correlation coefficient value is *r* = 0.71.

Instead, we focused on the aspects of localization that were useful in the clinical context. B-lines normally extend from the top to the bottom of the image following a radial direction, hence the relevant parameter is the angular position of the B-line. As a result, we assessed spatial localization by comparing the angle at which maximum attention was learned to the ground truth angle from expert annotators. To that end, images were converted to polar coordinates as shown in Figure 7, top. In polar representation, the mean attention over depth was computed for each angle, yielding a 1D attention vector that indicated which angle received more attention from the network, as shown in Figure 7, bottom.

**Figure 8:**
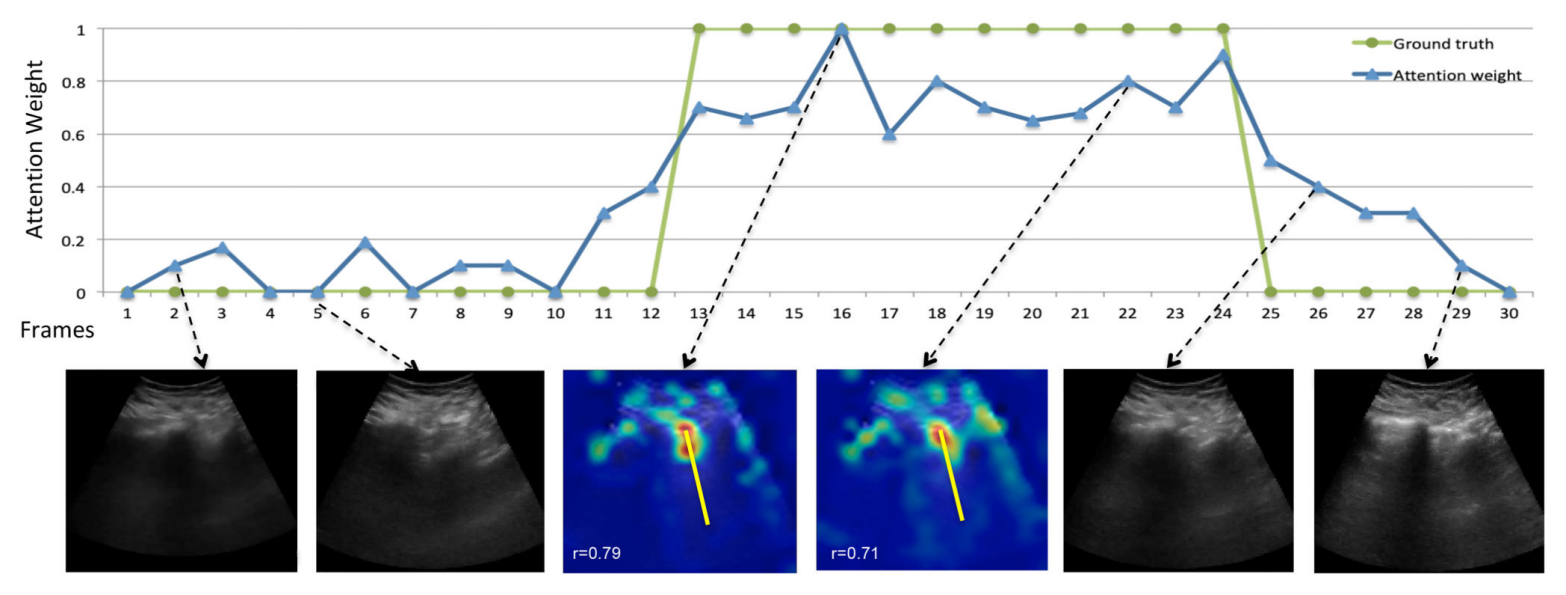
Generated temporal (horizontal axis) and spatial (heatmap overlaid onto B-Mode B-line frames) attentions estimated by our model on an example of an LUS video that includes both B-line and non-B-line frames. The top graph shows the temporal attention weights (in blue) and the corresponding ground truth annotations (in green). Spatial attention maps are visualized for B-line frames (for example frames 16 and 22): the yellow lines show the manual B-line annotations and, the correlation coefficient values (*r*), computed as described in the text, are presented at the bottom of each frame for illustration.

Then we employed the Pearson correlation coefficient [32] between the 1D attention vector and their related ground truth data, exemplified in Figure 7, centre row. This analysis showed that 1D attention vectors and their related ground truths are positively correlated, *r* = 0.67, *p* < 0.0001. Another example is shown in Figure 8, where both spatial and temporal (further evaluated in the next section) attentions are shown together.

To help interpret the quality of this correlation, Figure 6 shows an illustration of typical attention maps and the ground truth B-line location overlaid, suggesting that our spatial attention effectively attends to the B-line regions. These maps confirm the existence of more dominant features closer to the lung surface, where B-lines are generated.

### 5.3 Temporal B-Line Localization

The temporal localization is designed to find the B-line frames in the LUS video by predicting the importance of each frame for the classification task. We already showed that the temporal information improved the classification accuracy (Table 3). Additionally, temporal attention can discriminate frames that contain B-lines. To this end, Table 4 shows the IoU between the predicted B-line frames (using different thresholds) and the ground truth annotations, with an accuracy up to 69.7%. The calculation of the IoU is illustrated in Figure 8, top. To put the average accuracy into context, the example in the figure had an IoU of 78%. Furthermore, spatial attention maps are visualized for two B-line frames in the figure showing the ability of the model to localize B-line artefacts spatially and totally, being able to spatiotemporally localize B-line artefacts.

Additionally, a frame with the highest attention weight that is a representative frame with B-lines was identified on the test set with an accuracy of 88.6%. This could be useful to automatically provide clinicians with an exemplar frame to support the classification result.

### 5.4 Effect of Video Length on B-Line Classification Performance

Our B-line classification model (C2D+S+LSTM+T) was trained on the video lengths of one-second and was tested on the same video lengths with a performance over 80%. In a real clinical setting, however, the duration of the clips may vary (typically between 1 and 4 s). Additionally, the ability to use shorter videos can enable a more efficient translation to real time imaging. Therefore, we investigate the effect of various LUS video lengths on the model performance during the training and testing phases of the model.

The maximum length of our LUS videos is four seconds. As a result, we divided these four-second videos into shorter videos of length *l* = {1, 2, 3, 4} s. Results are presented in Table 5. Interestingly, when the model was trained on longer videos and then tested on shorter videos (For example, training = 4 s, and testing = 1 s), the performance decreased which means it needs more times for learning the video features. In contrast, training with shorter video lengths and testing with longer video lengths (For example, training = 1 s, and testing = 4 s) showed that one second of the four-second videos is enough for the model to find meaningful features during the testing. Further, when the same video length is used during the training and testing, the model performance is, in all cases, >83%.

### 5.5 Ablation Study

We performed an ablation study to investigate the contribution of the spatial and temporal attention mechanisms. The results are shown in Table 6.

As a baseline, we used the model without attentions, that is, a CNN feature extractor followed by an LSTM C2D+LSTM, with a performance (*F*1) of 79.7%. Adding spatial attention (C2D+S+LSTM) improved performance to 80.5%. The baseline with only temporal attention (C2D+LSTM+T) further improved performance to 81.0%, suggesting that temporal localization contributes more to performance than spatial localization. The spatiotemporal attention model (C2D+S+LSTM+T) outperformed all the others, with a top performance of 83.2%.

We also analyzed the performance with the spatial attention layer inserted after the first (C2D+S+LSTM+T*) or the second maxpool (C2D+S+LSTM+T), obtaining, respectively, 82.7% and 83.2% *F*1 scores, and suggesting that attention contributes more to classification accuracy when added later.

## 6 Discussion and Conclusions

We have proposed a spatiotemporal attention based convolutional+LSTM model capable of detecting the B-line artefacts and localizing them both spatially and temporally within LUS videos. Besides boosting the B-line classification accuracy, our spatiotemporal mechanism can increase the model interpretability, by suggesting when and where B-line artefacts are found. This localization ability was demonstrated quantitatively and qualitatively, despite being trained in a weakly-supervised manner. The model showed a B-line classification *F*1 score of 83.2% and a B-line spatial and temporal localization accuracy of *r* = 0.67% and IoU = 69.7%, respectively. These results are consistent with qualitative analysis via visual inspection of the resulting attention maps, which highlight frames and regions with the most salient B-lines in the video. In the context of competing methods, as outlined in Section 2, our method achieves state-of-the-art accuracy, and additionally can identify the most relevant frame. Furthermore, compared to a study by Gullet et al. [34], which investigated the agreement between physicians for assessing B-line presence on ultrasound in emergency patients and found intraclass correlation coefficients (ICC) agreement of 0.729, the results of our study for B-line classification (*F*1 score of 83.2%) is promising for a correct diagnosis.

A limitation of this study is that we focused on B-line artefacts only. Although this is the most important artefact in lung assessment, a complete lung examination including other features, such as pleural effusion, consolidation and confluent B-lines, will be incorporated in future work.

Our proposed model is a first step towards bringing automated LUS analysis to the ICU, particularly for the management of dengue patients in low and middle income countries where operator expertise is limited.

## Figures and Tables

**Table 1:** Details for spatial attention (N: number of output channels, K: kernel size, S: stride, P: padding, BN: Batch Normalization).

Index	Operation
(1)	Conv (N = 16, K = 3, S = 1, P = 1, BN, Relu)
(2)	Conv (N = 8, K = 3, S = 1, P = 1, BN, Relu)
(3)	Conv (N = 1, K = 3, S = 1, P = 1, Sigmoid)

**Table 2:** Overview of our LUS video dataset properties.

Resolution	Frame Rate	Video Length	No Patient	Annotation
640 * 480	30	About 5 h in total (avg 5 min per patient)	60	Frame-level & line annotation

**Table 3:** B-line classification performance. All the values are in percentages.

Model	Precision	Recall	F1
C2D+S+T	58.1	61.9	59.9
C3D+S+T	74.1	83.2	78.3
**C2D+S+LSTM+T**	77.2	90.3	**83.2**

**Table 4:** B-line localization performance at different IoU *α*’s. All the values are in percentages.

IoU	*α* = 0.1	*α* = 0.2	*α* = 0.3
C2D+S+T	37.7	32.4	27.6
C3D+S+T	65.1	62.3	52.5
**C2D+S+LSTM+T**	**69.7**	64.1	53.9

**Table 5:** B-line classification performance (*F*1) using different video length *l* during training and testing phases. All values are presented in percentages.

	Length *l* (s)	Testing			
		1	2	3	4
**Training**	1	83.2	83.0	82.8	82.6
	2	81.1	83.0	82.5	82.2
	3	79.7	80.1	83.6	83.8
	4	78.6	79.2	80.4	83.5

**Table 6:** Ablation experiments of the proposed model (C2D+S+LSTM+T). All the reported values are in percentages.

Model	Precision	Recall	F1
C2D+LSTM	75.1	85.0	79.7
C2D+S+LSTM	75.7	86.1	80.5
C2D+LSTM+T	76.1	86.8	81.0
C2D+S+LSTM+T*	76.8	89.8	82.7
**C2D+S+LSTM+T**	77.2	90.3	**83.2**

## Data Availability

The data presented in this study are available on reasonable request from the corresponding author.
